# Investigating the Neural Control of Social Behavior in *Drosophila ⁠melanogaster* Using a Low-Cost Optogenetics System

**DOI:** 10.59390/001c.147378

**Published:** 2025-12-30

**Authors:** Eric D. Hoopfer, Aaron Heidgerken-Greene

**Affiliations:** 1 Neuroscience Program Carleton College; 2 Department of Physics and Astronomy Carleton College

**Keywords:** optogenetics, Drosophila, social behavior, aggression, courtship

## Abstract

Optogenetics offers a powerful tool for students to explore how neural circuits generate behavior. Here we introduce a lab module using *Drosophila* to help students understand the principles of optogenetics through the study of social behaviors such as aggression and courtship. In this activity, students used the red-shifted opsin CsChrimson to activate P1 neurons—key regulators of male courtship and aggression. They observed that P1 activation elicits courtship in the absence of appropriate sensory cues and induces a persistent internal state that enhances aggression or courtship depending on social context, illustrating how a single neuronal population can regulate opponent behaviors and internal states. We implemented this module using FlyRig, an inexpensive, modular, open-source system we developed that provides precise light stimulation and synchronized video recordings suitable for automated tracking and classification tools. This lab module introduces students to the principles of optogenetics, experimental design, and quantitative behavior analysis, and provides a framework for exploring the neural basis of social behaviors and the internal states that drive them. Assessment of student experiences supports the utility of this lab activity in enhancing understanding of conceptual and experimental methods for studying neural control of behavior with optogenetics, as well as the usability of the FlyRig system for behavioral experiments.

Optogenetics has transformed the study of the neural basis of behavior by enabling precise, temporally controlled manipulation of genetically defined neuronal populations using light (Deisseroth, 2011). This technique involves expressing light-sensitive microbial opsins in targeted neurons, allowing neural activity to be modulated in awake, behaving animals. For example, channelrhodopsin, a light-gated cation channel originally isolated from the green alga *Chlamydomonas reinhardtii* (Nagel et al., 2003), depolarizes neurons in response to blue light (Boyden et al., 2005). Optogenetics enables millisecond-scale control of neural activity, facilitating causal investigations of how specific neural circuits contribute to behavior, perception, and neurological and psychiatric disorders (Fenno et al., 2011).

*Drosophila melanogaster* offers many advantages for implementing optogenetics in an undergraduate classroom setting due to its extensive genetic toolkit, which allows for optogenetic manipulation of specific neural circuits using simple genetic crosses. In *Drosophila*, the GAL4/UAS binary expression system is used for targeted expression of transgenes in defined neuronal populations (Brand & Perrimon, 1993). This system uses cell-specific promoters to drive expression of the yeast transcriptional activator GAL4, which binds to the upstream activating sequence (UAS) to initiate transcription of a gene of interest. A large, publicly accessible collection of GAL4 driver lines (Dionne et al., 2018; Jenett et al., 2012) is available for targeting specific circuits, as are a range of UAS-opsins transgene lines for neuronal activation (Klapoetke et al., 2014; Pulver et al., 2009) or inhibition (Mohammad et al., 2017). Many of these tools are available at little to no cost through the *Drosophila* research community and the Bloomington Drosophila Stock Center. In addition, open-source tools for automated fly tracking and behavior scoring (Eyjólfsdóttir et al., 2014; Kabra et al., 2013; Pereira et al., 2019) provide an unbiased and reproducible means of analyzing behavioral data across student groups, facilitating collaborative inquiry. These features make *Drosophila* an ideal system for implementing hands-on, inquiry-based neuroscience activities using optogenetics to help students understand how neural circuits give rise to behavior.

Red-shifted opsins such as CsChrimson, which are activated by red light that readily penetrates the adult cuticle (Inagaki et al., 2013; Klapoetke et al., 2014), make it possible to perform optogenetic experiments in freely behaving adult *Drosophila*. This expands the range of behaviors accessible for study in teaching labs and avoids the need for invasive preparations. Undergraduate lab courses have leveraged this approach to examine the neural control of escape responses, locomotion, and gustatory perception (Fu et al., 2023; Titlow et al., 2015; Vilinsky et al., 2018). Here, we describe a lab activity that uses *Drosophila* optogenetics for students to investigate the neural basis of social behaviors—specifically, male aggression and courtship. These behaviors consist of a stereotyped set of actions (Chen et al., 2002; Hall, 1994) that students can quickly learn to recognize, and which can be scored with machine-learning-based classifiers (Chowdhury et al., 2021; Dankert et al., 2009; Kabra et al., 2013; Yadav et al., 2025). Recent work has advanced our understanding of the neural circuits that underlie male aggression and courtship, including the sensory inputs that trigger them, motor pathways that produce them, and neuromodulatory systems that regulate internal states like motivation and arousal (Auer & Benton, 2016; Hoopfer, 2016; Lee & Wu, 2020). This well-studied circuitry provides an excellent framework for helping students understand how specific neuronal populations generate complex behaviors.

A key node in the circuitry that regulates male social behavior is the male-specific P1 neuron population, which integrates female sensory cues and promotes courtship and aggression. Optogenetic activation of P1 neurons elicits male courtship behaviors such as wing song, even in the absence of females (Bath et al., 2014; Inagaki et al., 2014). In addition to triggering immediate behavioral responses, P1 activation also induces a persistent internal state of social arousal that promotes aggression and courtship depending on social context (Hoopfer et al., 2015; Jung et al., 2020). Thus, optogenetic activation of P1 neurons can provide students with a compelling demonstration of how neural circuits mediate both acute behaviors and longer-lasting, state-dependent behaviors.

A major barrier to implementing optogenetics in the classroom is the cost of research-grade equipment, which typically includes machine vision cameras, optomechanical parts, and other expensive components (Inagaki et al., 2013; Robie et al., 2017). To address this, we developed FlyRig, an affordable, open-source platform that combines optogenetic stimulation with synchronized video acquisition. Each setup costs around $350 to build, supports parallel recording of multiple flies, and is compatible with widely used computational tools for automated tracking and behavior scoring, such as FlyTracker (Eyjólfsdóttir et al., 2014) and JAABA (Kabra et al., 2013). The system can be constructed and operated with minimal technical expertise, making it accessible to instructors and students alike. FlyRig complements existing low-cost solutions for fly optogenetics (Chagas et al., 2017; Pulver et al., 2011; Tadres & Louis, 2020) by enabling simultaneous recording of multiple fly pairs with software that runs on standard desktop operating systems.

Here, we describe a neuroscience laboratory module in which students use FlyRig to investigate the neural basis of social behavior in *Drosophila* through optogenetics. Students activate P1 neurons with CsChrimson and assess the effects on male courtship and aggression, illustrating how neural circuits regulate behavior on different time scales and integrate external cues to generate flexible behavior. The activity reinforces core neuroscience concepts, including neural circuit function, internal states, and molecular genetic techniques for modulating neural activity, while providing hands-on experience in hypothesis testing, experimental design, and quantitative behavioral analysis. By combining affordability with ease of use, FlyRig reduces barriers to incorporating optogenetics into undergraduate neuroscience curricula.

## MATERIALS AND METHODS

Instructional materials, including lab protocols, scoring guides, example videos, an instructor guide with a timeline and flow chart of the lab activity, and complete build and operation instructions for the FlyRig system, are provided in the Supplemental Materials.

### Implementation of Lab Activity

This optogenetics lab exercise was implemented as part of the laboratory component of *Foundations in Neuroscience*, an introductory course at Carleton College with no prerequisites and a typical enrollment of 30–40 students. The primary learning goals of the lab activity were to introduce the principles of optogenetics, deepen students’ understanding of how neural activity gives rise to complex behaviors, introduce students to quantitative analysis of animal behavior, and provide experience designing experiments to test hypotheses about neural circuit function.

Before the lab, students were introduced to gene expression, neuronal cell biology, and electrical and chemical signaling through in-class units. As pre-lab work, students watched videos on optogenetics (Nature Video - *Method of the year 2010: Optogenetics*), and how to use the FlyRig system (Supplemental Materials), and completed a pre-lab quiz. After the lab activity, students had a week to complete a post-lab written assignment in which they produced a graph and figure legend of their results and wrote a summary of the results. For additional context about how this laboratory fits into the lab course, we include the lab syllabus in the Supplemental Materials.

**Table 1.** Male aggression and courtship behaviors that students learned to identify. Behaviors with an asterisk were scored in example movies.

**Aggressive behaviors**	
Chasing	Male rapidly pursues the other fly.
Wing threat*	Male briefly charges forward with both wings thrust forward and then raises its wings at a 45-degree angle.
Lunging*	Male rears up on its hind legs and snaps down onto the other fly.
Tussling/Boxing*	Two males rise onto their hind legs to grapple with each other and may box with their forelegs.
	
**Courtship behaviors**	
Orienting & Following	Male orients its head towards the other fly and will start following it.
Tapping	Male extends a foreleg and touches the abdomen of the other fly.
Wing extension* (wing song)	Male extends one wing perpendicular to his body and circles around the other fly, while using the wing to produce courtship song.
Licking	Male extends its proboscis to touch the genitalia of the other fly.
Attempted Copulation*	Male will mount the other fly from behind, but falls off after a brief time (seconds).
Copulation*	Mounting lasts for several minutes. Flies can move together as one.

**Figure 1.**
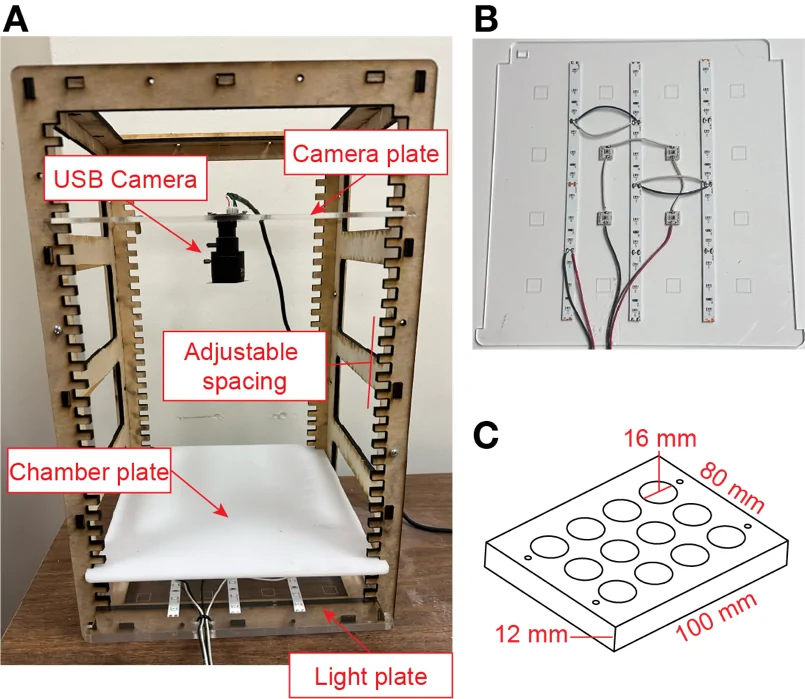
FlyRig experimental hardware. (A) Six laser-cut wood panels comprise the photostimulation frame. The rig contains a plate with the stimulation and infrared LEDs (light plate), one that diffuses light and holds the behavior chamber (diffuser plate), and one that holds the USB camera (camera plate). The relative placement of the plates can be easily adjusted in the frame. (B) The light plate contains four high-intensity LEDs for optogenetic stimulation wired in series and three infrared LED strips wired in parallel. (C) Schematic of acrylic behavioral chambers used for social behavior assays.

The lab activity began with a 30-minute lecture reviewing the principles of optogenetics and the use of *Drosophila melanogaster* as a model for studying social behavior. Students then spent approximately 30 minutes learning to recognize a subset of male aggression and courtship behaviors (Table 1). They reviewed written descriptions and example video clips of each behavior. They then practiced identifying these behaviors in videos of male-male pairs where neurons previously shown to elicit male social behaviors are being thermogenetically activated with dTRPA1 (Hoopfer et al., 2015). The video set included one line that selectively promoted wing threat (Line 1), one that triggered the full repertoire of aggressive behaviors (Line 2), one that evoked wing extension oriented toward the other fly (Line 3), and one that elicited undirected wing song (Line 4). After scoring the videos, students compared their results to ground-truth annotations and discussed their observations as a class to build consistency in behavioral identification.

In the main component of the lab (2-3 hours), students worked in small groups (3–4 students), each with their own FlyRig system. For a class of ~30–40 students, one photostimulation rig per 5–6 students allows multiple groups to work in parallel while keeping costs manageable and minimizing bottlenecks in the lab workflow. Students were asked to characterize the behaviors elicited by optogenetic activation of P1 neurons, without prior instruction about the role of these neurons in male social behavior. The goal was for students to generate conclusions from their experiments about how P1 neurons regulate courtship and aggression. To become familiar with the FlyRig system, each group first conducted a 20-minute pilot experiment, observing the behaviors of solitary males from each genotype in response to light pulses at different frequencies. After a class discussion, students selected a stimulation protocol (1 minute of 30 Hz pulsed light) and then tested both solitary males and male pairs from each genotype. Students manually scored the number of lunges and wing extensions during light-on and light-off periods, graphed their results, and presented preliminary findings to the class. Based on these initial observations, each group formulated a hypothesis regarding P1 neuron function in male social behavior and designed a follow-up experiment to test it.

**Figure 2.**
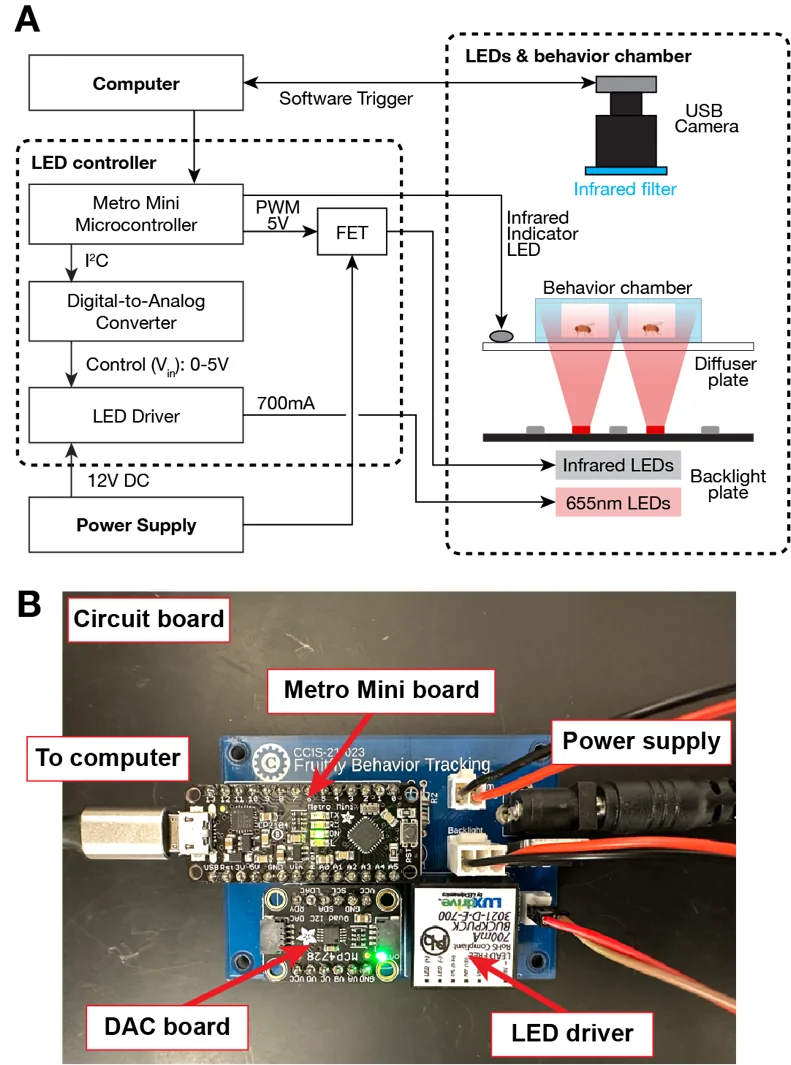
Light control board. (A) Schematic of light control and video recording circuit. (B) Assembled circuit board.

### FlyRig equipment and software

**Figure 3.**
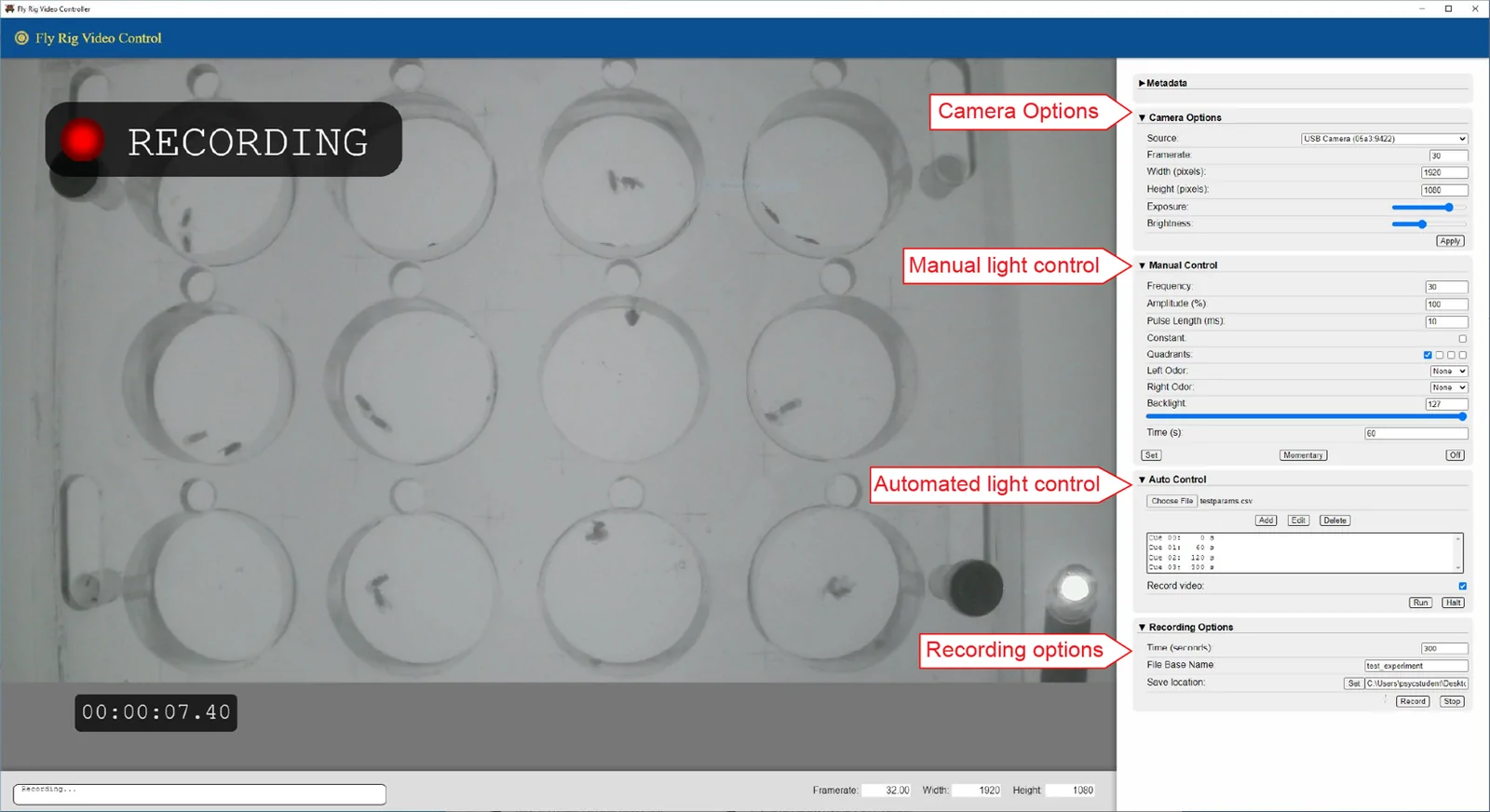
FlyRig Software. FlyRig software controls photostimulation and records videos of fly behavior. A central window provides a view of the recording. The camera resolution, frame rate, exposure, and brightness can be adjusted in the “Camera Options” window. The stimulation LED and infrared LEDs can be manually controlled in the “Manual Control” window. A set duration of light stimulation can be delivered with the “Set” button, or the user can momentarily turn on the light by clicking on the “Momentary” button. The “Auto Control” window allows users to create or upload a more complex sequence of light stimulations, which is synchronized to the start of the video recording.

The FlyRig system consists of three main components: the photostimulation rig (Figure 1), the LED/backlight circuit board (Figure 2), and integrated software (Figure 3).

Building and operating the system requires basic electronics assembly tools (e.g., soldering iron), access to a laser cutter, and a computer to run the FlyRig software and store video recordings. Laser-cut parts can be fabricated using local makerspaces, university facilities, or small commercial services that accept CAD files for custom orders (e.g., Ponoko.com). However, no specific vendor is required, allowing flexibility based on availability and cost.

The photostimulation rig (Figure 1A) consists of a frame into which light, diffuser, and camera plates are inserted. The parts were laser cut from the following materials: frame panels (3/16” thick nominal plywood), light and camera plates (0.125” clear acrylic; McMaster-Carr 8560K191), and diffuser plate (0.125” white Delrin; McMaster-Carr 8573K13). The frame can also be cut from 0.2” acrylic or Delrin to aid in cleaning.

High-intensity deep red LEDs (655nm wavelength, Luxeon Star LEDs SR-05-D0000) were mounted on the light plate for photostimulation, along with strips of 850nm IR LEDs (ledlightsworld.com 5M-HK-8MM-F3528-850-30-NW-IR-12) to visualize fly behavior (Figure 1B). The fly behavior chamber (Figure 1C) was placed on the diffuser plate, and fly behavior was recorded using a mini-USB camera with a 2.8-12mm zoom lens (ELP ELP-USBFHD06H-FV) mounted on the camera plate. A long-pass IR filter (B&H Camera 87CP3) was affixed to the lens to block light from the high-intensity LEDs.

We adapted the LED control circuit from Pulver et al. (2011) and Inagaki et al. (2013), and mounted all components on a custom-printed circuit board (PCB; JLCPCB.com; Figure 2). Design files for the PCB and instructions for assembling the circuit on a standard breadboard are available for download (Supplemental materials). A Metro Mini microcontroller, controlled by Arduino code, regulates both the timing and intensity of photostimulation. To control the stimulation LEDs, the microcontroller sends digital commands via the I²C (Inter-Integrated Circuit) protocol to a digital-to-analog converter (DAC), which outputs a 0–5V analog control voltage. This analog signal drives an externally dimmable constant-current LED driver (Buckpuck 3021-D-E-700; maximum output 700 mA), powered by a 12 V DC supply. For the infrared (IR) backlight LEDs, brightness is modulated independently using a 5V PMW signal from the microcontroller to a field-effect transistor (FET) configured as a low-side switch. This setup enabled real-time adjustment of IR illumination to optimize video contrast and image quality. To mark the timing of light stimulation in video recordings, we mounted an additional IR indicator LED near the behavioral chamber. A separate digital output from the microcontroller, synchronized with the stimulation protocol, controlled the indicator LED, providing a visible timing reference in the recorded footage. We also developed an expanded version of the PCB capable of driving four sets of LEDs and eight solenoid valves independently (Supplemental Materials).

FlyRig software (Figure 3) was developed in JavaScript using Electron (electronjs.org) and Node.js (nodejs.org), and runs on both Windows and macOS operating systems. It features an integrated graphical user interface for controlling LED activation (manually or automatically) and IR lighting, logging experimental metadata, and recording videos in h264 mp4 format. The GUI can be used to record video without the circuit board connected. To facilitate classroom use, a precompiled executable version is available for Windows, allowing the software to be run without installation or code modification.

### Fly stocks

The following fly stocks were used: *pBDPGAL4U* (BDSC #68384; Pfeiffer et al., 2010), *R15A01-p65⁠.⁠AD(attP40);GMR71G01-GAL4DBD(attP2)* (referred to as P1a in text; Hoopfer et al., 2015), *20XUAS-IVS-CsChrimson.mVenus(attP2)* (BDSC #55136; Klapoetke et al., 2014). We are happy to provide all stocks on request. Fly stocks were maintained at room temperature (21-25^o^C) in bottles with Jazz-Mix Drosophila Food (Fisher, AS153) and transferred to fresh bottles every 2 weeks.

### Optogenetic experiments

For optogenetic experiments, *GAL4* males were crossed with *UAS-CsChrimson* virgin females in vials containing fly food supplemented with 0.4 mM all-trans-retinal (Sigma-Aldrich, #R2500). Crosses were maintained in constant darkness at 25°C and 50–60% humidity, with parents transferred to fresh retinal food vials every 3–4 days. Male progeny were collected 0–2 days post-eclosion and housed in groups of 15–20 flies per vial for 6–7 days on retinal food under constant darkness. Flies were transferred to fresh retinal food vials one day before behavioral experiments.

Optogenetic experiments were conducted as described in Hoopfer et al. (2015). Experiments took place in an acrylic behavior chamber consisting of 12 cylindrical arenas, each measuring 12 mm in height and 16 mm in diameter (Figure 1C). A thin layer of food (apple juice with 2.5% sucrose and 2.25% agar) was applied to the bottom plate to stimulate aggression (Lim et al., 2014). Arena walls were coated with Fluon (PTFE-30; Tarheel Ants), and the top plate was treated with Sigmacote (Sigma, SL2) to prevent flies from climbing. The diffuser plate was positioned approximately 4 cm above the light plate. The maximum irradiance measured with an optical power meter (Thorlabs, PM160) with constant light is approximately 0.07mW/mm^2^. Single males or male pairs of the same genotype were loaded into the chambers via aspiration and allowed to acclimate for 5 minutes. Experiments were conducted at room temperature under ambient lighting conditions. Behavior was recorded at 30 frames per second with a resolution of approximately 25 pixels/mm.

### Behavioral analysis and statistics

Student-generated movies were processed after class using Caltech FlyTracker (Eyjólfsdóttir et al., 2014), which tracks body and wing positions, and JAABA-based classifiers (Hoopfer et al., 2015; Kabra et al., 2013) to identify bouts of lunging and unilateral wing extension (i.e., wing song). Statistical analyses were conducted in MATLAB. Mann-Whitney U tests were used to compare behaviors between genotypes. Statistical significance was defined as α < 0.05.

### Student survey

After the laboratory activity, students were asked to complete an attitudinal survey regarding the conceptual and technical aspects of the activity. Completion of the survey was optional, and no personal identifying information (gender, class, year, major, etc.) was collected as part of the survey. The study was reviewed and approved (exemption granted) by the Institutional Review Board of Carleton College.

**Figure 4.**
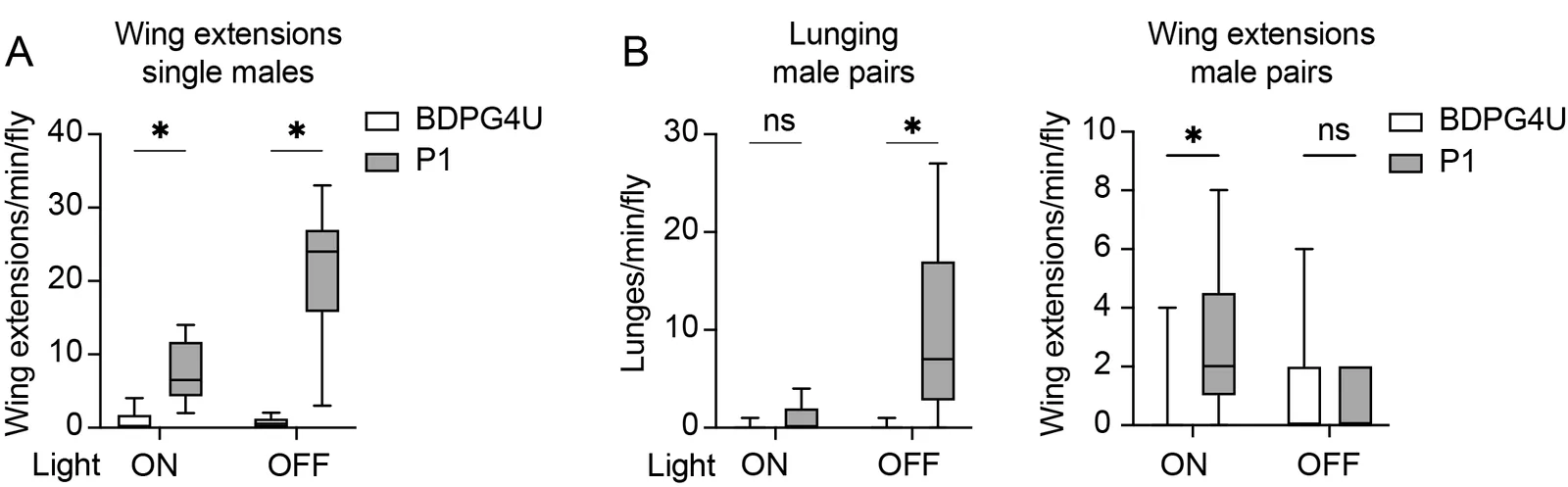
Automated behavior scores of student data from optogenetic activation of P1^a^ neurons. *BDPG4U>CsChrimson* (open bars) or *P1^a^>CsChrimson* (filled bars) males were photostimulated for 1 minute with 655 nm light at 30 Hz, followed by 2 minutes of no light. (A) Frequency of wing extensions by solitary males during photostimulation (ON) and after photostimulation (OFF). n = 7 & 6 flies for BDPG4U & P1^a^, respectively. (B) Frequency of lunging (*left*) and wing extensions (*right*) by pairs of males during and after photostimulation. n = 7 pairs for BDPG4U and P1^a^. Boxplots show the median (line) and 25-75% of the data (box) with whiskers spanning 5-95% of the data. Asterisks represent p-value < 0.05 from a Mann-Whitney U-test comparing *BDPG4U* and *P1^a^* males.

## RESULTS

### Optogenetic stimulation of P1 neurons in male flies

Students investigated the role of P1 neurons in controlling male social behavior by optogenetically stimulating P1^a^ neurons—a subset of 8–10 neurons in the P1 cluster previously shown to regulate male courtship and aggression (Hoopfer et al., 2015). Working in groups, students addressed two questions: (1) What behaviors are promoted during and after neuronal stimulation? and (2) Does the presence of another male alter these behaviors? Behavioral responses of *P1^a^/CsChrimson* males (referred to as P1^a^) were compared to genetic control males (*BDPG4U/CsChrimson,* referred to as BDPG4U) (Pfeiffer et al., 2010) that do not express GAL4 in the nervous system. Students manually scored wing extensions (courtship) and lunges (aggression) during class, and student video recordings were later processed by the instructor for automated analysis of the same behaviors (Figure 4).

To characterize the behaviors elicited by P1^a^ activation, students first tested solitary males. Compared to genetic controls, P1^a^ males exhibited a significantly elevated rate of unilateral wing extension both during and after photostimulation (Figure 4A). Students then repeated the assay using pairs of males to assess the influence of social context on P1^a^-evoked behaviors. As in the solitary condition, wing extension was significantly elevated during the stimulation period relative to controls (Figure 4B). However, unlike single males, P1^a^ pairs exhibited a marked increase in lunging following stimulus offset, indicating a transition from courtship to aggression in the post-light period (Figure 4B).

Based on these observations, students developed hypotheses about how P1 neurons regulate male social behavior. One class of experiments tested whether the behaviors displayed depended on the activation threshold of P1 neurons by varying the intensity or frequency of light stimulation. A second class of experiments examined whether persistent post-stimulation behaviors resulted from a fly-intrinsic change (i.e., an altered internal state) or from external influences. For example, students tested whether a *P1^a^/CsChrimson* male would still display aggression toward a non-aggressive *BDPG4U/CsChrimson* male to determine whether sustained aggression was due to escalating attack-counterattack cycles between two P1^a^-activated males (i.e., social feedback). In summary, P1^a^ activation produces robust, context-dependent behavioral phenotypes that students used as a basis for designing follow-up experiments, providing additional practice in experimental design, data analysis, and interpretation while reinforcing their understanding of how neural circuits control behavior.

### Student Feedback

We conducted a voluntary survey of students enrolled in two sections of our introductory neuroscience laboratory course (n = 17 responses; response rate = 53%) to evaluate their perceptions of both the pedagogical and technical components of the lab activity (Table 2). The majority of respondents agreed that the lab enhanced their understanding of how channelrhodopsin is expressed in specific neurons and how it functions to elicit action potentials.

**Table 2.** Student evaluation of conceptual and technical aspects of the optogenetics laboratory. Student responses to queries ranked on a Likert scale (1 = Strongly Disagree, 2 = Disagree, 3 = Somewhat Disagree, 4 = Somewhat Agree, 5 = Agree, 6 = Strongly Agree). Mean and standard deviation of responses are shown. *n* = 17 students.

**Question**	**Mean**	**SD**
I am confident in my ability to explain how channelrhodopsin works to evoke action potentials.	5.1	±0.7
I am confident in my ability to describe how the channelrhodopsin gene is expressed in specific neurons in fruit flies.	4.9	±0.9
I am confident in my ability to formulate a hypothesis and design an experiment to test how neurons influence behavior.	5.3	±0.8
I am confident in my ability to identify appropriate measures and controls for a behavioral experiment.	5.1	±0.8
This lab increased my interest in neuroscience research.	4.7	±1.3
It was easy to make a video recording using the FlyRig software.	5.4	±0.6
It was easy to control the light stimulation using the FlyRig software.	5.3	±0.7
The FlyRig software worked reliably.	4.6	±1.3
Overall, using the FlyRig software to conduct an experiment was easy.	5.0	±0.7

Students also reported increased confidence in formulating hypotheses about the neural control of behavior and in designing experiments to test these hypotheses. On the technical side, students found it straightforward to record videos and control light stimulation using the FlyRig system, and generally agreed that the system functioned reliably. One group reported difficulty connecting to the camera; however, this was resolved by unplugging and reconnecting the device to reset the USB port. Overall, students found the FlyRig software easy to use for conducting experiments.

## DISCUSSION

Optogenetics is a widely used tool in neuroscience, making it important for students to gain direct experience with its principles and applications. *Drosophila melanogaster* offers an accessible platform for optogenetics in teaching laboratories and has been used to introduce students to synaptic physiology and the neural basis of behavior (Fu et al., 2023; Pulver et al., 2011; Titlow et al., 2015; Vilinsky et al., 2018). This lab activity extends these approaches by engaging students in circuit-level investigations of social behaviors, specifically aggression and courtship, which are shaped by sensory cues from conspecifics, prior social experience, and internal states (Jiang & Pan, 2022; Lee & Wu, 2020; Schretter, 2025). These features make them a rich model for exploring how neural circuits regulate complex behaviors while reinforcing core concepts in optogenetics, genetic targeting of specific neurons, and hypothesis-driven experimental design. Post-lab surveys indicated that the activity enhanced student understanding of both the conceptual basis and experimental methods for studying neural control of behavior using optogenetics.

Optogenetic activation of P1^a^ neurons produces a robust and easily recognizable phenotype that can be used to illustrate various concepts related to neural circuit control of behavior. Students observe that stimulation of P1^a^ neurons triggers courtship even without appropriate sensory cues (e.g., no female present or even the presence of a male), demonstrating that P1 neuron activity is sufficient to initiate courtship behavior. The persistence of behavior after stimulation prompts students to consider whether P1 neurons induce internal states, such as arousal, motivation, or drive, that sustain behavior over time (Anderson, 2016; Berridge, 2004). Finally, the differences in post-activation behavior that depend on social context (courtship when alone, aggression in the presence of another male) illustrate how external cues are integrated with internal states to shape behavioral output (Kennedy et al., 2014). These observations help students understand how neural circuits orchestrate behavior across different time scales and social contexts, while providing a foundation for generating and testing new hypotheses. Lastly, studying the neuronal basis of social behaviors such as aggression and courtship encourages students to view these behaviors through a biological lens. Post-lab assigned readings, such as Anderson & Adolphs (2014), which provides an accessible overview of how emotions can be studied in animals, can help students connect their findings to broader concepts in neuroscience and psychology.

This lab activity can be modified in several ways to fit different pedagogical goals. We have used this activity for a 2-hour summer high school program by omitting the experimental design module at the end. In this shortened version, students still learn about optogenetics, observe it in action, and learn to interpret neural gain-of-function experiments and develop hypotheses about how neurons control behavior. Conversely, there are several ways to expand the activity. First, although the instructor processed student videos in the activity described here, students can readily perform automated behavior scoring and statistical analyses themselves. Incorporating this step can provide valuable training in coding, image analysis, and quantitative data analysis. To support this, we provide instructions and code for video processing, fly tracking, and automated behavior scoring (Supplemental Materials). Second, this module can be extended into a Course-Based Undergraduate Research Experience (CURE). For example, in our introductory neuroscience course, students spend the final three weeks of the term building on this experiment by designing and conducting a research project investigating P1 neuron function in social behavior. Past projects have explored how neuromodulators such as dopamine shape P1-induced courtship and aggression, whether P1 activation is rewarding to male flies, and how internal states such as hunger, sleep deprivation, or stress influence P1 excitability.

Recent advances in mapping the neural circuits underlying *Drosophila* social behavior in both sexes (Asahina, 2018; Auer & Benton, 2016; Oren-Suissa & Shirangi, 2025; Schretter, 2025) provide additional opportunities for classroom experiments on aggression and courtship. Several GAL4-based driver lines available from the Bloomington Drosophila Stock Center target neurons that promote specific behaviors upon activation. For example, TK5fa-GAL4 (BDSC#51975) promotes male aggression (Asahina et al., 2014), 26E01-GAL4 (BDSC#60510) promotes female aggression (Palavicino-Maggio et al., 2019), and 60G08-GAL4 (BDSC#39260) promotes aggression in both sexes (Chiu et al., 2021). Other lines target specific aggressive behaviors, such as 20E08-GAL4 (BDSC#59851), which elicits wing threat (Duistermars et al., 2018). For courtship, fru-GAL4 (BDSC#66696) labels ~2,000 neurons in the male brain and promotes multiple courtship actions (Inagaki et al., 2013; Pan et al., 2011), while 22D03-GAL4 (BDSC#49871) promotes wing song (Robie et al., 2017). By selecting different driver lines, instructors can tailor the module to target a variety of social behaviors, broadening the range of experimental questions students can explore.

### FlyRig system

FlyRig builds on earlier low-cost systems (Pulver et al., 2011; Vilinsky et al., 2018) by integrating software that enables students to easily adjust light stimulation parameters and timing, record videos and experimental metadata, and test multiple fly pairs simultaneously. The resulting data are compatible with automated behavior scoring tools such as FlyTracker and JAABA, facilitating quantitative analysis of student-generated data. JAABA-based classifiers for several male aggression and courtship behaviors (Chowdhury et al., 2021; Yadav et al., 2025) are already available and can be used with this system. Compared to other systems such as PiVR (Tadres & Louis, 2020) and FlyPi (Chagas et al., 2017), which integrate optogenetic stimulation, video recording, and tracking using a Raspberry Pi platform that allows standalone operation, FlyRig runs on Windows or macOS. This can make it more straightforward to implement in labs with existing computers and provides a familiar interface for students and instructors. One current limitation is that, unlike PiVR and FlyPi, which perform tracking natively, FlyTracker currently requires MATLAB since no standalone executable is yet available. However, FlyRig movies should be compatible with other open-source tracking systems are such as Ctrax (Branson et al., 2009), SLEAP (Pereira et al., 2019), and ToxTrac (Rodriguez et al., 2018).

The system’s simple, modular design allows for a range of experimental applications. Automated control of light stimulation ensures reproducibility across trials, while manual triggering of stimulation light allows students to carry out rudimentary “closed-loop” experiments where stimulation is paired with a specific behavior or sensory cue in real time. For example, students have used this feature to test whether P1^a^ activation can induce conditioned place preference by photostimulating males only when they entered one side of an arena. Easily interchangeable LED plates allow quick switching between experiments using different wavelengths, including neuronal silencing with halorhodopsin or archaerhodopsin. Additionally, we provide plans for an expanded circuit board (Supplemental materials) capable of controlling four sets of LEDs independently. This can be used to control different wavelength LEDs on the same plate, allowing for activation and inhibition experiments using the same hardware. We also used it to control LEDs in different quadrants of a behavioral arena, enabling real-time place preference assays (Shao et al., 2017) to test whether flies prefer regions where neurons are optogenetically activated. This versatility allows multiple behavioral assays to be conducted using the same hardware platform.

In conclusion, this lab module demonstrates an approach to integrate circuit-level investigations of social behavior in *Drosophila melanogaster* into undergraduate neuroscience education. By pairing *Drosophila*’s genetic toolkit with the FlyRig system, students gain hands-on experience with optogenetics while investigating how neural activity shapes behavior in a context-dependent manner. FlyRig’s simple, low-cost, and modular design lowers barriers to implementing optogenetic experiments, making them accessible to educators and researchers in a variety of settings. As circuit-level maps of *Drosophila* behavior continue to expand, tools like FlyRig will help translate cutting-edge neuroscience into accessible, inquiry-based learning experiences for students.

### SUPPLEMENTAL MATERIAL

*Supplemental file 1.* Instructor guide with a timeline, flowchart and step-by-step instructions for lab activity, a class syllabus, an example class worksheet, and student handouts.

*Supplemental file 2*. Behavior example clips, male-male pair videos with and without annotations, and behavior timestamps.

FlyRig hardware and software files, including build instructions and user manuals, can be downloaded from https://github.com/hoopfere/FlyRig/wiki.

### Address correspondence to:

Dr. Eric D. Hoopfer, Neuroscience Program, 1 N College St., Carleton College, Northfield, MN 55057. Email: ehoopfer@carleton.edu

Copyright © 2025 Faculty for Undergraduate Neuroscience

www.funjournal.org

## Supplementary Material

Supplemental file 1

Supplemental file 2
