# Supplementary material for: Investigating the Neural Control of Social Behavior in Drosophila ⁠melanogaster Using a Low-Cost Optogenetics System
Source: J Undergrad Neurosci Educ. 2025 Dec 30;24(1):4–14. doi: 10.59390/001c.147378 (PMC13127674; doi:10.59390/001c.147378)

**Table of Contents**

[Building and using FlyRig](#_Toc213588856)

[Instructor resources](#_Toc213588857)

[Equipment and reagents](#_Toc213588858)

[Fly stocks](#_Toc213588859)

[Fly rearing and handling](#_Toc213588860)

[Social behavior assay](#_Toc213588861)

[FlyRig hardware](#_Toc213588862)

[Software](#_Toc213588863)

[Timeline for Optogenetic Lab Activity](#_Toc213588864)

[Flowchart of lab activity](#_Toc213588865)

[Lab Procedure](#_Toc213588866)

[Preparing flies for experiments](#_Toc213588867)

[Making retinal food](#_Toc213588868)

[Fly crosses & collection](#_Toc213588869)

[Preparing a fly pooter and loading flies](#_Toc213588870)

[Procedure: Making the fly pooter](#_Toc213588871)

[Procedure: Loading flies](#_Toc213588872)

[Preparing apple juice agar food](#_Toc213588873)

[Preparing social behavior assay](#_Toc213588874)

[Processing videos for automated scoring](#_Toc213588875)

[Worksheet: Optogenetics – Altering neural activity with light](#_Toc213588876)

[Handout: Drosophila Social Behaviors Descriptions](#_Toc213588877)

[Example Syllabus: Foundations in Neuroscience Laboratory](#_Toc213588878)

# Building and using FlyRig

Up-to-date build instructions, software code, user guides, and teaching materials can be found at <https://github.com/hoopfere/FlyRig/wiki>.

# Instructor resources

- Equipment and reagents
- Timeline for preparing the lab activity
- Flowchart of the lab activity
- Lab procedures
- Example worksheet
- Example Syllabus for lab course
- Fly Behavior Descriptions handout
- Video clips of male social behaviors (Supplemental file 2)
- Behaviors movies (Supplemental file 2)
  - Student movies
  - Timestamps of behaviors in each movie
  - Annotated movies

# Equipment and reagents

## Fly stocks

- All stocks listed below are available upon request from Eric Hoopfer ([ehoopfer@carleton.edu](mailto:ehoopfer@carleton.edu)). Stocks with a BDSC# can be purchased from the Bloomington Drosophila Stock Center (<https://bdsc.indiana.edu>).
- *20XUAS-IVS-CsChrimson.mVenus(attP2)* (BDSC #55136; referred to as *UAS-CsChrimson*)
- *pBDPGAL4U* (BDSC #68384; referred to as BDPG4U)
- *R15A01-p65.AD(attP40);GMR71G01-GAL4(attP2)* (referred to as P1^a^)
- *R71G01-GAL4(attP2)* (BDSC #39599) *and R15A01-GAL4(attP2)* (BDSC #48670) produce the same behavioral phenotype with optogenetic actiation as *P1^a^*, and thus, can be substituted for those flies in this exercise.

## Fly rearing and handling

- 100 mg *all-trans* retinal (Sigma-Adlrich R2500). Makes approximately 750 ml of 400µM retinal food.
- Narrow fly vials and plugs (Fisher AS515 & AS273)
- Jazz-Mix fly food (AS153, Fisher)
- Paintbrushes for fly sorting
- Stereo dissecting microscope and light source for fly sorting
- Fly anesthesia system (CO₂ or ice)
- Fly pooters (Tygon tubing 1/4” ID x 3/8” OD, 1000 ul pipette tips, cotton)

## Social behavior assay

- Apple juice
- Agar
- Sucrose
- Behaviors chambers: Four chambers can made from one 12x12 in sheet of 0.25 in thick clear acrylic for the chamber body (McMaster-Carr [8560K354](https://www.mcmaster.com/8560K354)), two 12x12 in sheets of 0.125 in thick clear acrylic for the chamber top and bottom plates (McMaster-Carr [8560K239](https://www.mcmaster.com/8560K239)), and eight 6-32 screws for securing chamber top. Dfx files for laser cutting the chambers can be downloaded from [FlyRig/wiki](https://github.com/hoopfere/FlyRig/wiki).
- Lab tape for securing chamber bottom.
- Fluon (PTFE-30; Tarheel Ants) for coating walls.
- Sigmacote (Sigma, SL2) for coating top plate.

## FlyRig hardware

- Computer running Windows or Mac OS with FlyRig software installed and two available USB ports for the camera and circuit board.
- FlyRig hardware and circuit board. Parts list, lasercutter templates, printed circuit board plans, and complete build instructions found at the [FlyRig/wiki](https://github.com/hoopfere/FlyRig/wiki).

## Software

- FlyRig software (source code and Windows executable version) found at [FlyRig/wiki](https://github.com/hoopfere/FlyRig/wiki)
- Arduino IDE software ([download](https://support.arduino.cc/hc/en-us/articles/360019833020-Download-and-install-Arduino-IDE))
- Automated behavior analysis software (requires MATLAB)
  - multiCropVideos.m ([FlyRig/wiki](file:///Users/ehoopfer/Library/CloudStorage/GoogleDrive-ehoopfer@carleton.edu/My%20Drive/Manuscripts/OptoRig%20MSS/Supplemental%20Material/Supplementary%20Material/FlyRig/wiki)) – creates individual movie of each behavior chamber for FlyTracker
  - FlyTracker (<https://kristinbranson.github.io/FlyTracker/>)
  - JAABA ([https://jaaba.sourceforge.net](https://jaaba.sourceforge.net/)) – executable versions available for Windows and Mac OS

# Timeline for Optogenetic Lab Activity

**Note:** The following timeline was modified from McKellar and Wyttenbach, “A Protocol Demonstrating 60 Different Drosophila Behaviors in One Assay.” J Undergrad Neurosci Educ. 2017 Jun 15;15(2):A110–A116. PMID: 2869043. Please refer to their paper for detailed instructions for setting up a basic fly workstation, fly handling, stock maintenance, and performing fly crosses. Below, I outline the basic steps and specific tasks for optogenetic experiments.

Timeline (assumes flies are maintained at ~22°C)

**Week –12:** Order fly stocks.

**Week –8:** Expand *UAS-CsChrimson* into several vials. Transfer (flip) *BDPG4U* and *P1^a^* (*15A01AD-71G01DBD*) to new vials; keep the original vial for 2 more weeks as backup.

**Week –6:** Expand *UAS-CsChrimson* for virgin collection (aim for ~10 vials). Flip *BDPG4U* and *P1^a^* to new vials.

**Week –4:** Collect *UAS-CsChrimson* virgin females for crosses.

**Week –3:** Cross *UAS-CsChrimson* virgins with *BDPG4U* and *P1^a^* males on retinal food. Prepare two replicate crosses per student group.

**Week –2:** Remove (discard) parents from vials.

**Week –1**: Collect experimental males, housing ~20 per retinal food vial. Provide each student group with 2 vials each of *BDPG4U/UAS-CsChrimson* and *P1^a^/UAS-CsChrimson.* Flip males to fresh retinal vials 2–3 days before the experiment. Prepare fly pooter and make apple juice agar food and store at 4°C.

**Day of activity:** 30–60 min before the lab, prepare assay plates for behavior experiments.

**After activity:** Video processing and tracking*

**Next day:** Process tracked videos with JAABAPlot and analyze data*

*****(see <https://github.com/hoopfere/FlyRig/wiki/Tracking-Instructions> for instructions)

# Flowchart of lab activity

Approximate time spent on each module of the lab activity (in bold).


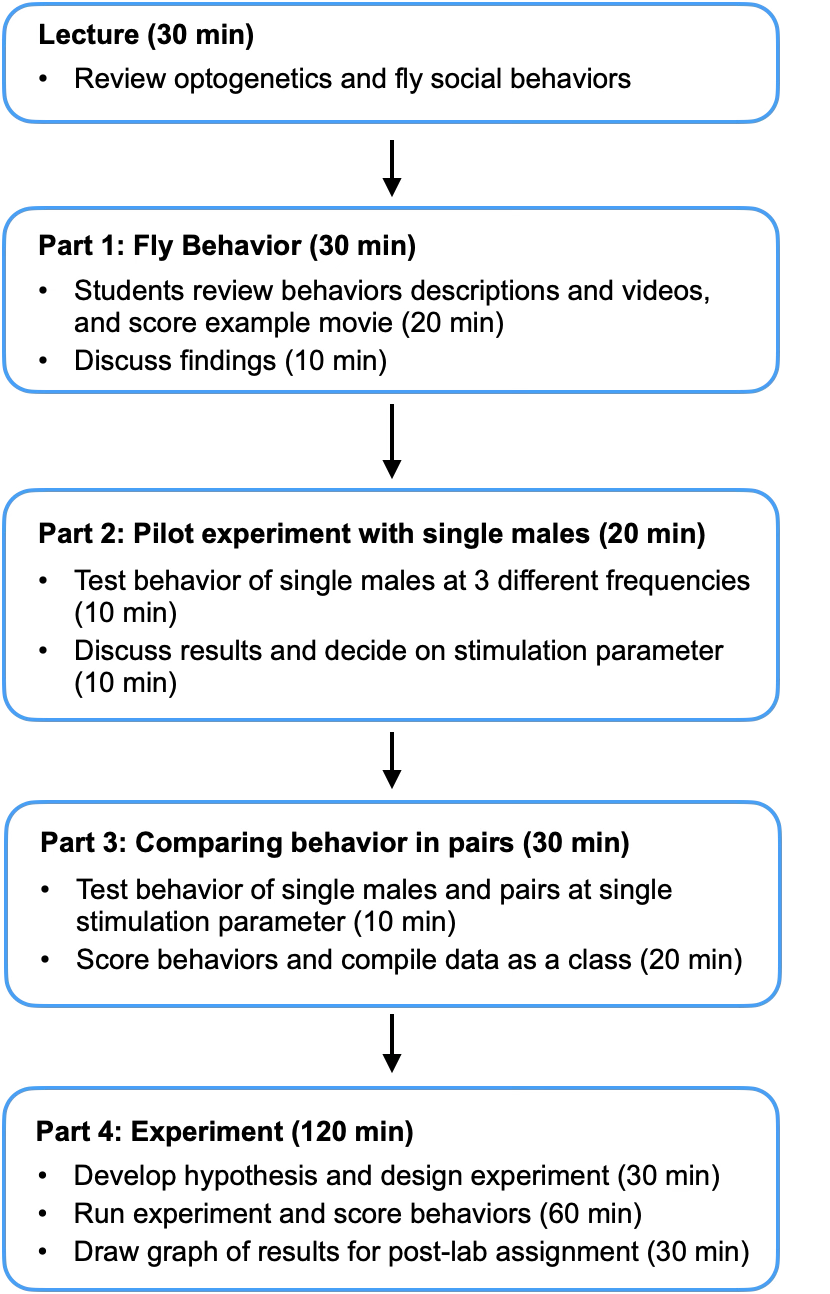


# Lab Procedure

## Preparing flies for experiments

### Making retinal food

The following protocol will make 25 vials for 400 µM all-trans retinal (ATR) food. ATR food vials should be protected from light and last about 2 weeks when stored at 4^o^C.

ATR stock: Make a 100mM solution of all-trans retinal (ATR) in 95% ethanol. Wrap ATR solutions in aluminum foil to protect from light and store in a -21C freezer.

ATR vials

- Prepare 250ml of Jazz-Mix fly food according to product instructions.
- Let food cool below 60^o^C. Stirring it occasionally.
- Add 1 ml of 100 mM ATR to food and stir until completely mixed.
- Use an automated pipettor to add ~ 10 ml of food to each vial.
- Loosely cover vials with aluminum foil or a dark cloth to protect from light and allow them to cool completely (overnight is recommended). Do not put vials in refrigerator to speed cooling as this will produce condensation which shortens the lifespan of the food.
- Cap vials with plugs once they are cooled. Cover with aluminum foil and store at 4^o^C.

### Fly crosses & collection

1. Collect virgin females of the *UAS-CsChrimson* stock. To facilitate virgin collection, remove flies from the bottle the evening before collecting. Collect the next morning and again in the evening, if necessary. Repeat this until you have sufficient virgin females.
2. Collect GAL4 males on the day you set up the crosses.
3. Add 6-8 virgin females and between 4-6 males in a 400uM ATR food vial. Some dry yeast can be sprinkled into the vial to facilitate egg laying.
4. Cover vials with aluminum foil and keep at room temperature or in a 25^o^C incubator if one is available.
5. To increase yield, transfer parents to fresh vials every 3–4 days (up to twice), yielding ~3 vials of progeny per cross.
6. Discard parents from final set of vials 3-4 days after the last flip.
7. Progeny will begin to emerge ~12-14 days later at room temperature (21-23^o^C) and ~10 days later at 25^o^C.
8. One week prior to experiments, collect male progeny between 1-2 days old. Progeny will begin to emerge ~12-14 days later at room temperature (21-23^o^C) and ~10 days later at 25^o^C. House no more than 20 males in each ATR food vials for one week. Keep vials covered with aluminum foil.

## Preparing a fly pooter and loading flies

*Notes:*

- Gentle transfer of flies using aspiration is the preferred method for behavioral assays. Anesthetizing with CO_2_ adversely affects behaviors for up to a week after anesthesia. Cold anesthesia is acceptable but can also adversely affect social behaviors and memory.
- Transferring files into behavioral chambers should be done as gently as possible to prevent disruption to behavior.
- Transferring flies is tricky and requires some practice! Collect males from one of the UAS-CsChrimson vials (can be done when collecting virgin females) and transfer to food vial (~20 males per vial). Once the males have recovered from anesthesia, practice transferring them one by one into the behavior arenas. Don’t get discouraged if you have escapers at first. You’ll quickly get better with some practice.

*Hazards:*

- Keep the mouthpiece to your fly pooter clean! Hang it around your neck while using it instead of setting it down on a benchtop. Store in a ziplock back.
- Use caution when using razor blades to cut pipet tips! Cut tip against a flat surface and keep fingertips clear of the razor blade.


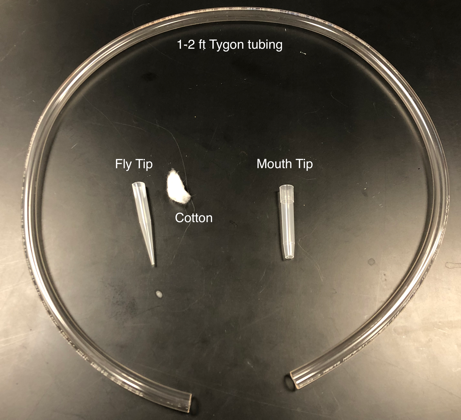


### Procedure: Making the fly pooter

1. Cut Tygon tubing to preferred length.
2. Trim the large end off of one pipet tip with a razor blade. This will be the tip you use to load flies (fly tip). Use a clean razor blade and place a paper towel on the benchtop to keep tips clean (one of them will go in your mouth eventually!
3. Insert a small ball of cotton into the large end of the fly tip. The cotton ball should be about the same diameter as the opening.
4. Insert the fly tip into the tubing.
5.
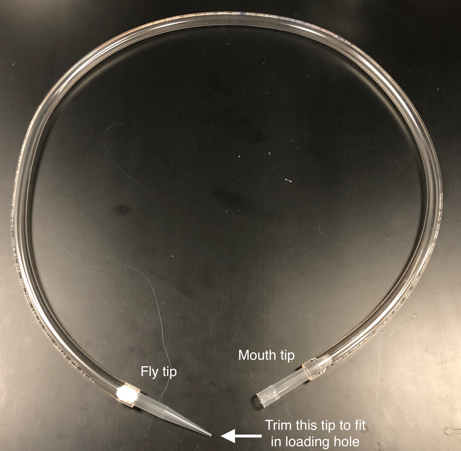
Cut the other tip about 1/3 the way up from the narrow end. This will be the tip you put in your mouth (mouth tip).
6. Place the mouth tip narrow side first into the other end of the tubing.
7. Trim the fly tip so that it is just a little larger than the size of a fly (~2 mm). If you trim too much off, you won’t be able to fit the tip into the loading holes of the behavior chamber (~3 mm). It might help to have a chamber nearby to check the fit.
8. You’re all set to start loading flies!


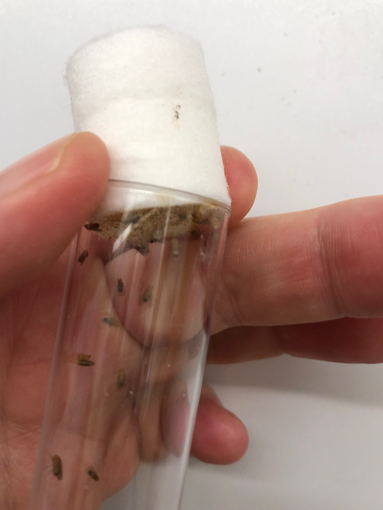

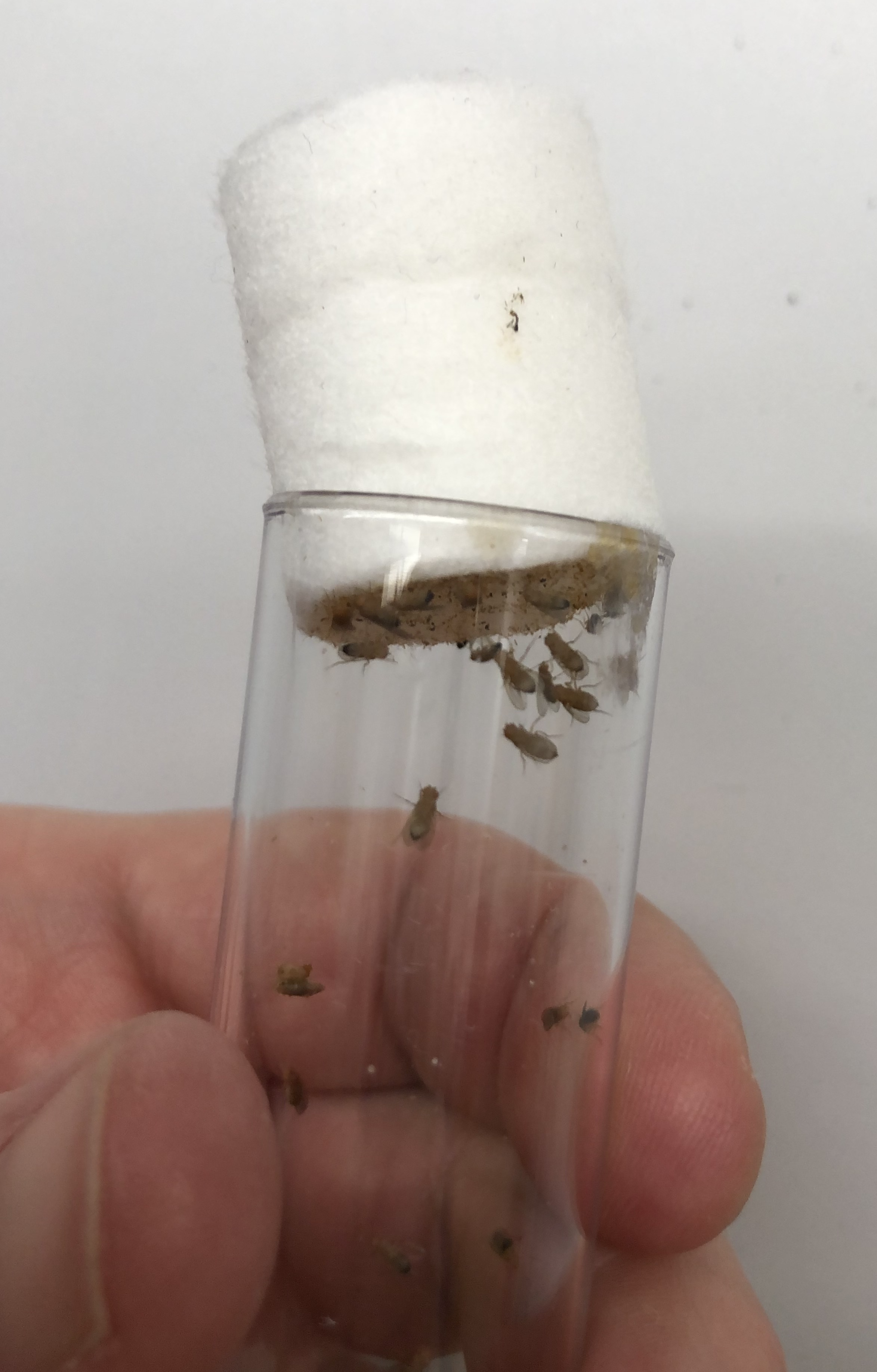


### Procedure: Loading flies

1. Holding the fly vial with one hand, gently loosen the plug so that one end is near the top of the vial. However, you don’t want to pull it out since flies will escape.

1. With the mouth tip into your mouth, insert the fly tip into the vial at the end where the plug is near the opening. Keep some downward pressure on the fly plug to keep the plug sealed and prevent flies from escaping.


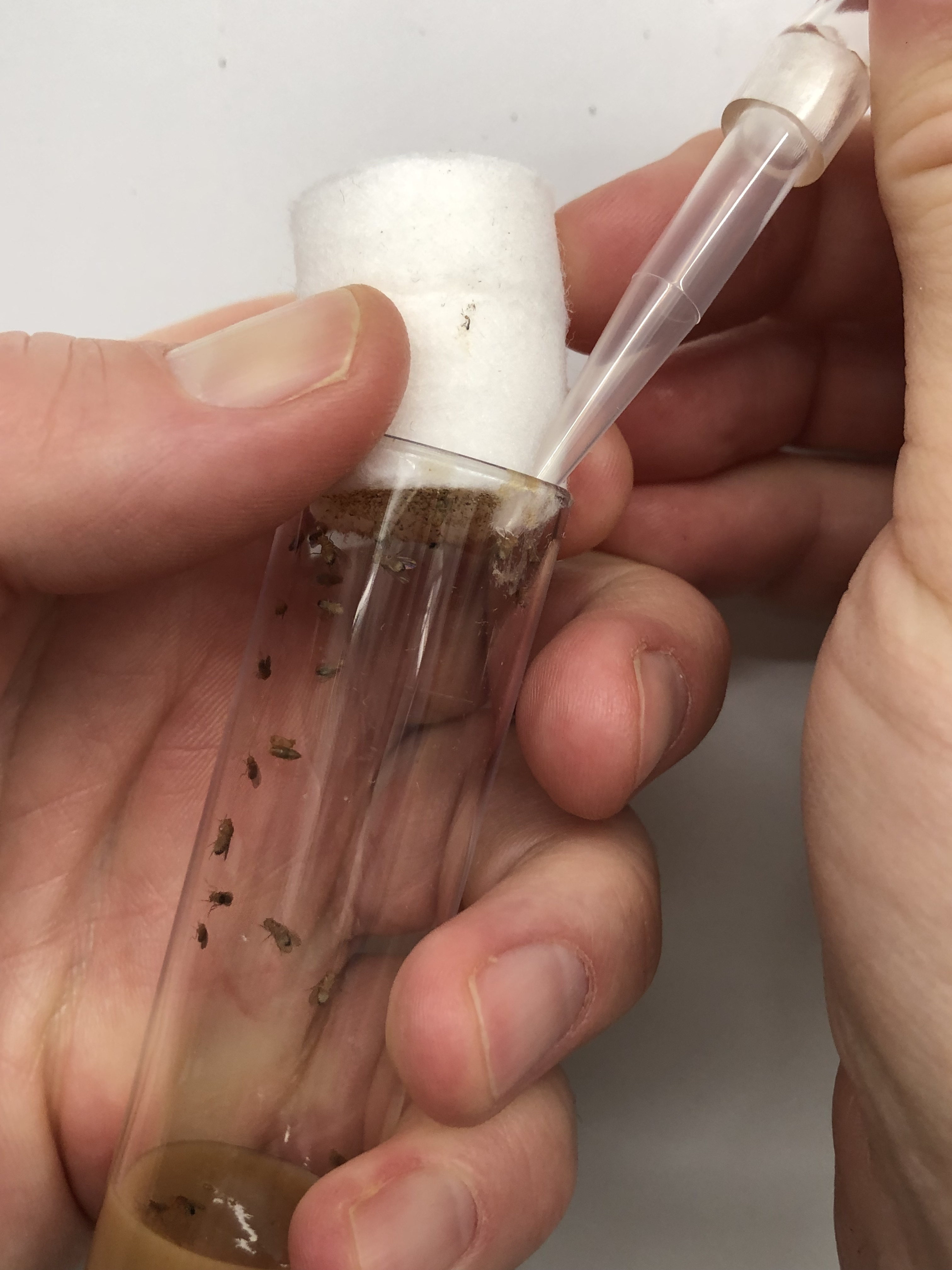

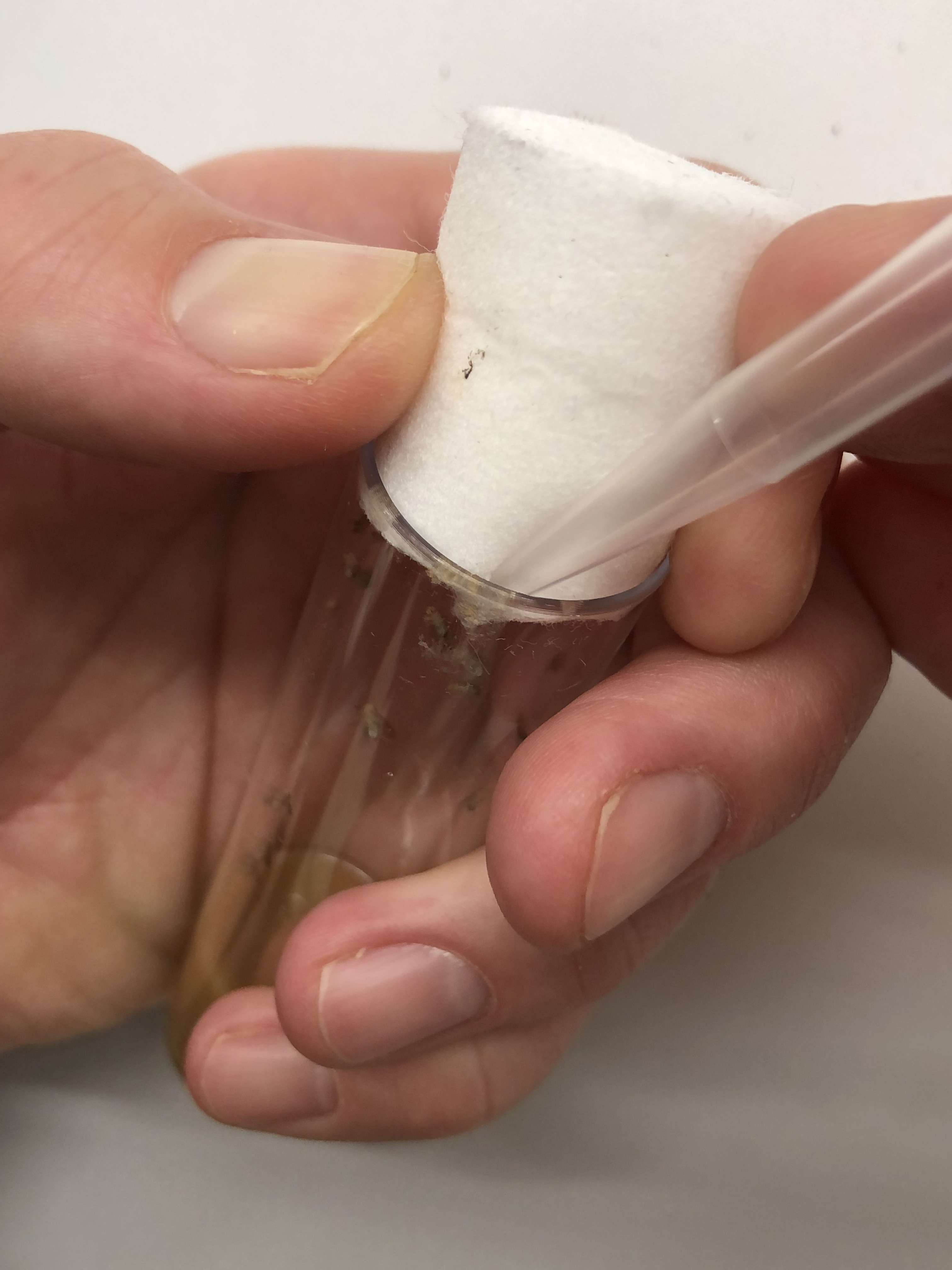


1. Flies will naturally crawl to the top of the vial where you can catch them. This behavior is known as negative-geotaxis (*geo* = Earth (Latin), *taxis* = directional movement (Latin) – movement against gravity.
2. When a fly nears the pipet tip, quickly apply suction to suck it into the tip.
3. Remove the tip and replace the plug.
4. You can prevent the fly from escaping the pipet tip by occasionally applying a little more suction or covering the hole with your finger.
5. Place the tip into the loading hole of the chamber and gently blow the fly out of the tip. If the fly isn’t going towards the opening of the tip, flick the tip or gently tap it on the table to knock the fly down and try loading again.
6. Once the fly is loaded, plug the loading hole with a yellow pipet tip to prevent the fly from escaping.
7. When all flies are loaded, slide another plate along the top of the chamber to cover holes as you remove the pipet tips.
8. Slide the top plate into place and tighten the thumbscrews.
9. You’re all set to run your assay.

## Preparing apple juice agar food

Notes:

- The chamber floor has a layer of apple-juice agar food, which provides food cues necessary for male aggression
- This recipe makes 200 ml of food, which is sufficient for ~6 assay plates. Food can be stored at 4^o^C for up to 2 weeks.

Procedure:

1. In the 100 ml flask, add 5g sucrose and 50 ml apple juice. Swirl to combine.
2. In the 250 ml flask, add 4.5g agar and 150 ml apple juice. Swirl to combine.
3. Microwave each solution until completely dissolved. **Caution: Can boil over!**
4. Use safety gloves or hot pads. Gently swirl the flask every 30 sec until you obtain a clear solution without any particles.
5. Pour sucrose-apple juice solution into the flask with agar solution and swirl to combine.
6. Melted food can be used now or stored at 4oC for later use.

##

## Preparing social behavior assay

- 1. Melt apple-juice agar food in a microwave until liquid.
  2. Place the bottom plate into a pouring tray (e.g., the lid of a 1000 ul pipet box or tissue culture plate) and pipet ~30ml of food on top of the plate. Gently push the plate down to displace any air bubbles under the plate and pop any bubbles with a pipette tip.
  3. Allow plates to cool for 30 min to 2 hours at room temperature.
  4. Cut around the plate with a razor blade and lift out the plate using a spatula. Wipe off any excess food on the bottom of the plate.
  5. Place food side up on the bottom of the chamber plate and secure with tape.
  6. Attach top plate and thumbscrews.
  7. Load males by aspiration (see [Preparing a fly pooter and loading flies](#_Preparing_a_fly)).
  8. Place behavior arena on diffuser plate of FlyRig photostimulation rig and allow them to acclimate for 2-5 minutes before beginning experiment.

## Processing videos for automated scoring

⚠️ For up-to-date instructions, please check

Part 1: Create movies for each behavior chamber

1. Launch Matlab
2. Make sure the multiVideoCrop.m function is in the Matlab Path
   1. MATLAB can only run functions that are either:
      - In the **current folder**, or
      - On the **MATLAB path**.
   2. To check your path, type path in the Command Window.
   3. You can add a folder to the path with:

addpath('C:\path\to\your\folder')

1. In the command window type:
2. Select the folder with movies to process.

⚠️ Note that the videos should all be the same (i.e., same number of chambers in the same relative positions).

1.
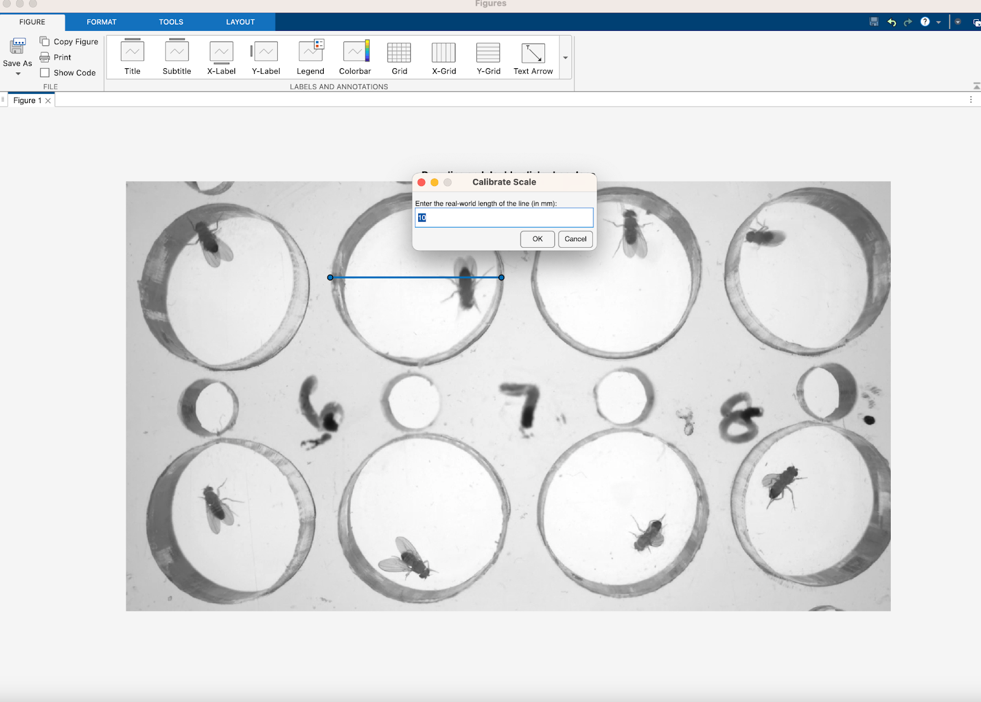
Drag line across a known distance (e.g., the diameter of a chamber) and enter the distance in mm.
2. Enter the configuration of the chambers
   1. Number of chamber (total)
   2. size (diameter in mm)
   3. Number of rows
   4. Number of columns
3. After you click OK, it will take a moment to compute a background image.
4. An image will appear with a frame from the movie with rectangles (ROIs) for each chamber overlaid. You should move the ROIs around until each fits properly around the chamber. The number on the ROI will be appended to the new movie.
5. Close window to move to the next movie.
6. The output of this function will be a movie (mp4) for each chamber with the number of the ROI.

Part 2: Running FlyTracker

ℹ️ Instructions for running FlyTracker can be found at: <https://kristinbranson.github.io/FlyTracker/userguide.html>

1. Launch FlyTracker in Matlab by typing tracker in the command prompt.
2. Select folder with movies to track.
3. Calibrator recommendations
   1. Works best by selecting “Automatically detect chamber”
   2. Leave “fixed” button unchecked
4. We usually track movies using parallel processing for speed. If an error is encountered during tracking, move the movie to a new folder and track later using only one core.
5. Tracks can be visualized using the visualizer function.

Part 3: JAABA automated behavior detection

ℹ️ Instructions for using JAABA can be found at: <https://jaaba.sourceforge.net>

1. Download behavior classifiers for lunging and wing extension from wiki
2. Classify behaviors and visualize data using JAABAPlot (<https://jaaba.sourceforge.net/PlottingResults.html#LoadingClassifiers>).

# Worksheet: Optogenetics – Altering neural activity with light

Despite its small size and the many differences from humans, the fruit fly *Drosophila melanogaster* has been fundamental to our understanding of biology and neuroscience. Fruit flies have been used to study everything from how genes direct brain development to how our internal biological clocks work. Although the fly brain contains about 100,000 neurons (compared to the ~100 billion in humans), fruit flies share the same basic building blocks of the nervous system—neurotransmitters, ion channels—and possess the same basic senses as humans. They are capable of complex behaviors and have been used to study the neurobiology of learning, addiction, sleep, anxiety, and social behaviors.

In today’s lab, you will use optogenetics to activate different populations of neurons in the brain of male flies and observe what social behaviors this induces. Neuroscientists use these types of experiments to understand how circuits of interconnected neurons in the brain control neural processes and behaviors.

The goals of this lab are to:

1. Explain how channelrhodopsin is used to activate neurons.
2. Identify social behaviors like courtship and aggression in the fruit fly, *Drosophila melanogaster*.
3. Design an experiment to test how neurons regulate behavior.
4.
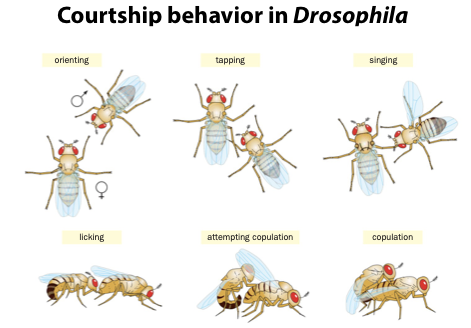
Plot and explain your data in a bar chart.


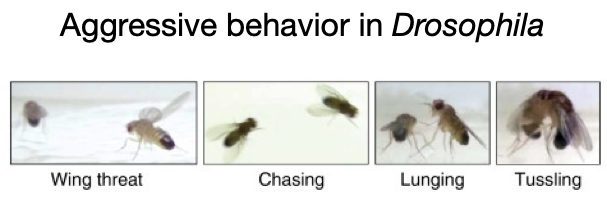


*Drosophila* male aggressive behavior

*Drosophila* male courtship behavior

**Part 1: Fly Behavior**

In the first part of this lab, you will familiarize yourself with different fly behaviors and how to identify them.

All files for this section are in a folder called, “Optogenetics Lab” on the desktop of your computer.

1. Open the file called, “Fly Behavior Descriptions.” This gives a brief description of what courtship and aggressive behavior look like in fruit flies. Read over these to familiarize yourself with the different behaviors.
2. Open the folder called “Fly Behavior Example.” This contains video examples of courtship and aggression behaviors. Study the examples and compare them to the descriptions in the “Fly Behavior Descriptions.” Your goal is to be able to recognize these behaviors when flies do them in your experiments.
3. Next, practice identifying the behaviors. Open the “Behavior movies” folder. This contains videos of optogenetic experiments. Each movies show a pair of male flies where a set of neurons in the male’s brain are being activated. In the table on the next page, write down what behaviors you observe and when they happen. Be specific about the behaviors (wing song, lunging, etc.). Refer back to the example videos and descriptions if you’re unsure.

| Movie | Behaviors | Start time (sec) | End time (sec) |
| --- | --- | --- | --- |
| Line 1 |  |  |  |
| Line 2 |  |  |  |
| Line 3 |  |  |  |
| Line 4 |  |  |  |

**Part 2: Optogenetic activation of fly neurons**

Next, you’ll use light to activate neurons in the brain of male flies and observe what behaviors are induced. You will test the following two lines:

1. Control: No channelrhodopsin (ChRs) expressed in the brain.
2. P1: Channelrhodopsin expressed in 8-10 neurons in the male brain.

**Pilot experiment**

1. You’ll be given a behavior chamber with single males of each genotype.
2. Start with the light stimulation conditions shown below. Give the flies 60 seconds of light. Record your observations in the table on the next page.
   1. **Frequency**: This controls the number of flashes of light per second
   2. **Amplitude**: This controls the intensity (or brightness) of the light (leave it at 100%)
   3. **Pulse** **length**: This controls how long each flash is (leave set to 10 milliseconds)
   4. **Quadrants**: Make sure the left-most box is checked


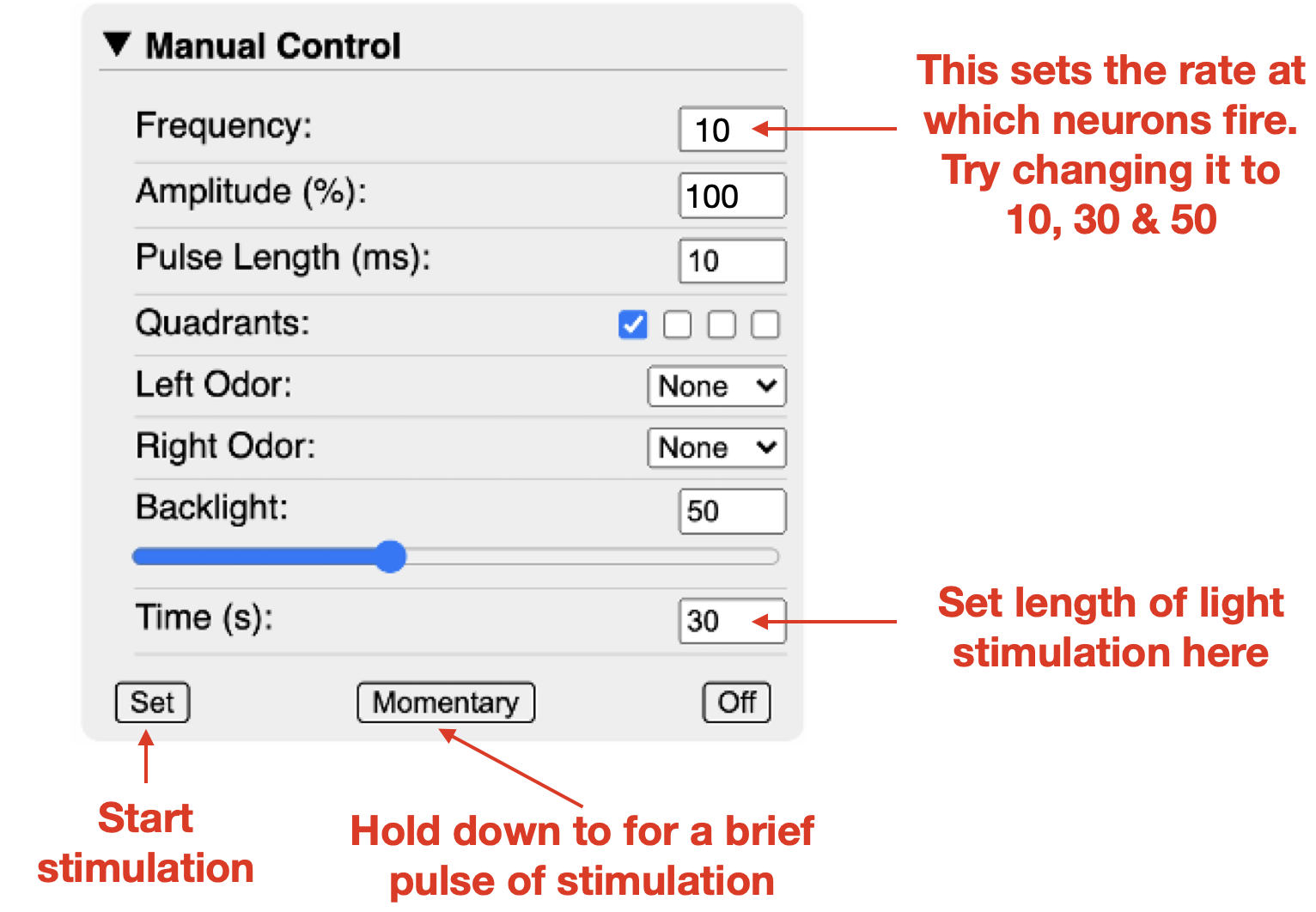


1. Try increasing or decreasing the frequency of light and see whether this changes the flies’ behavior. Record your observations in the table below.

| Line of flies | Observations  (Record Behaviors at each frequency) |
| --- | --- |
| Control |  |
| P1 males |  |

**Part 3: Comparing single males and pairs of males**

1. Next, you’ll use the light stimulation settings we agreed on based on our pilot experiments to optogenetically activate single males and pairs of males of each genotype.
2. You will receive a single male of each genotype and a pair of males of each genotype. Allow the flies to acclimate for 2 minutes before starting your experiment.
3. Record the behavior of the males during the 1 minute stimulation period and for 2 minutes after stimulation (i.e., video duration of at least 180 seconds).
4. When you’ve finished, ask the instructor for another round of flies. You’ll repeat this experiment twice.
5. After you’ve finished, score the number of lunges and wing extensions in each experiment in the table found on Moodle (look for your sheet with your group name). We’ll compile this data together as a class and graph the results.

**Part 4: Experiment**

Based on the results of your pilot experiment, design a follow-up experiment to investigate how P1 neuronal activity in influences male behavior.

1. What is your experimental question? What is your specific hypothesis?
2. Write out the methods for your experiment. Include the following:
   1. What will you manipulate (Independent variable)?
   2. What will you measure/count (Dependent variable)? Be specific as specific as you can.
   3. Sketch out a rough draft of what the graph of your expected results will look like.
3. When you’re ready to conduct your experiment, check in with your instructor. We will give you 3-4 pairs of each genotype to test.
4. Record your movies and save them to analyze later. It is also helpful to record any observations while doing the experiments. Use the rest of this page for that
5. **Draw** a graph of your results. The graph should show the mean +/- standard error of the mean. Make sure to label your axes.

**Male Courtship Behaviors in Drosophila**

**Orienting/Following:** Male fly orients its head towards the other fly and will start following it.

**Tapping:** Male extends a foreleg and touches the abdomen of the other fly.

**Singing:** Male extends one wing perpendicular to his body and circles around the other fly. The male is vibrating his wing up and down very quickly (difficult to see on camera) to produce a song that the other fly can hear.

**Licking:** Male fly extends its proboscis to touch the genitalia of the other fly.

**Attempting copulation:** Male will mount the other fly from behind, but falls off after a brief time (seconds to minute).

**Copulation:** Mounting lasts for several minutes. Flies can move together as one.


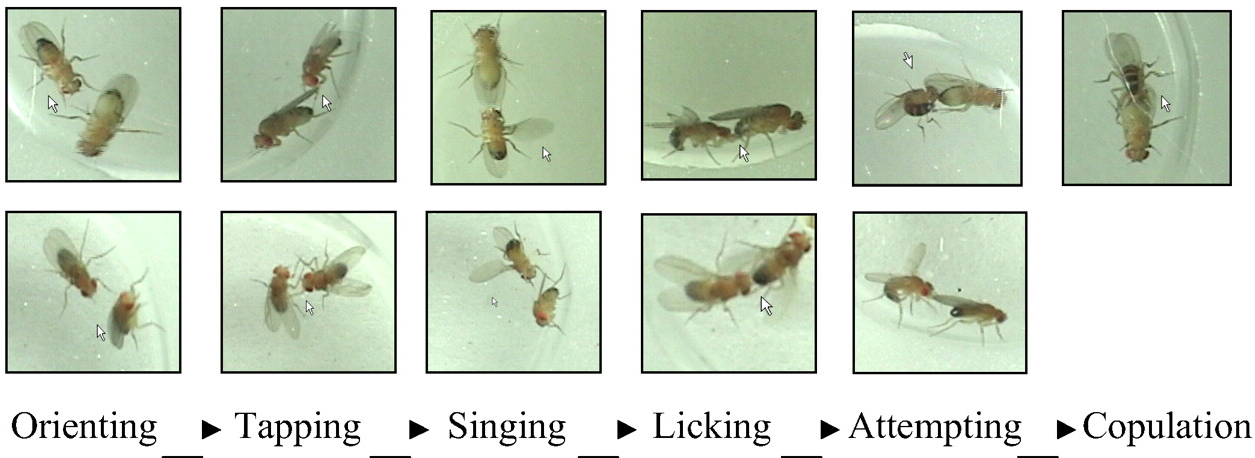


**Figure 1: Courtship behaviors seen from top and side views.** Images from Dai et al., (2008) The evolution of courtship behaviors through the origination of a new gene in *Drosophila.*

PNAS: 105 (21) 7478-7483.

# Handout: Drosophila Social Behaviors Descriptions

**Male Aggressive Behaviors**

**Lunging**: One fly rearing briefly on its hind legs and snapping down onto the other fly.

**Tussling:** Both flies grip each other with their front legs. The bodies face each other so that their axes of symmetry are parallel. Connected solidly in this configuration, they move about in jerks with high velocity and acceleration.

**Wing threat:** One ﬂy quickly raises **both** wings to a 45° angle towards opponent. Flies will often pump their wings out to 90 degrees while briefly charging towards the other fly.

**Figure 2: Male aggressive behaviors.** Images from Dankert et al., (2009) Automated monitoring and analysis of social behavior in Drosophila. Nat. Methods: 6(4):297-3

# Example Syllabus: Foundations in Neuroscience Laboratory

**Neur127**

**Eric Hoopfer & Sarah Meerts**

**Class Meeting: Hulings B04**

**Tues 1-5 PM, Wed 2-6 PM**

**COURSE DESCRIPTION**

The brain is the most important organ in your body. Not only is it responsible for orchestrating the physiological processes that ensure your survival, but it is also the physical source of what makes you, you. Understanding how this organ carries out these amazing functions is the ultimate goal of neuroscience. Neuroscience spans many fields of study, from genetics and molecular biology to computer science and psychology, and it borrows experimental approaches from all these fields. In this lab, you will learn to think like a neuroscientist and explore some of the experimental concepts and techniques that neuroscientists use to understand nervous system function.

**LEARNING GOALS**

By the end of this lab course, you will be able to:

- Describe and carry-out various experimental methods commonly used in neuroscience
- Interpret and critique primary scientific literature
- Effectively communicate scientific concepts and findings with scientific writing
- Design and perform experiments to investigate the neural basis of behavior, document experimental methods and results, and interpret newly acquired data
-
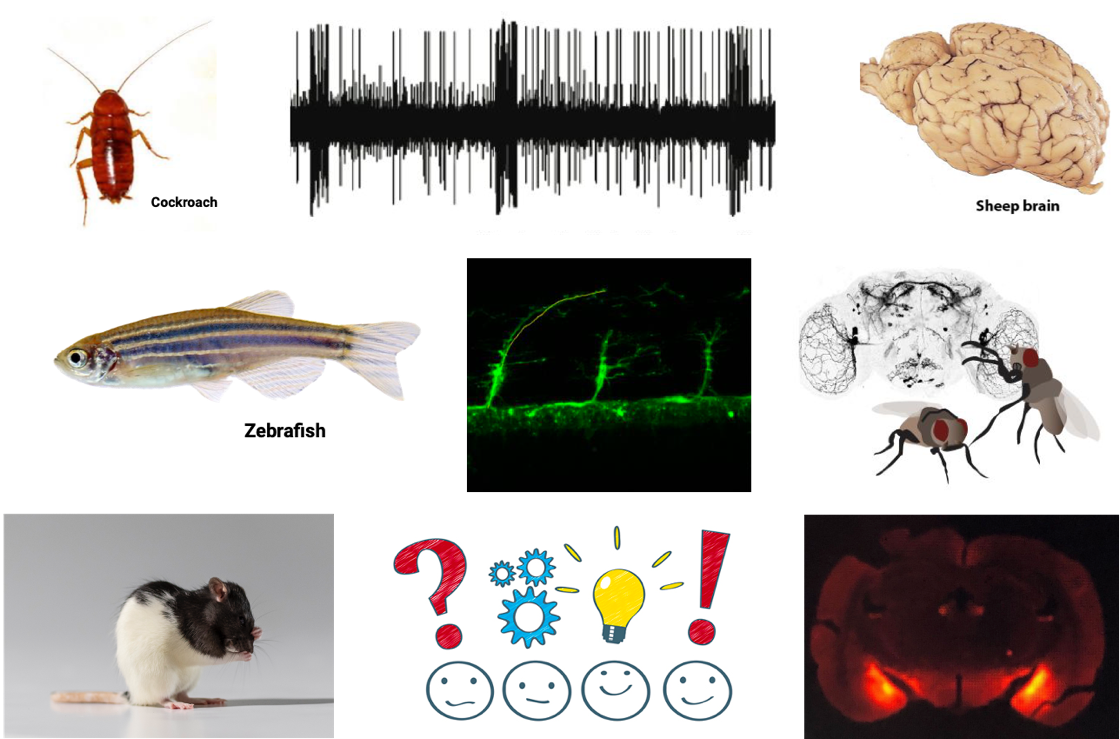


**COURSE WEBSITE**

On the Foundations in Neuroscience Lab Moodle site, you can find announcements, readings to prepare you for the lab, and discussion forums.

**ASSESSMENT**

|  | Percentage of grade |
| --- | --- |
| Pre-lab assignments | 10 |
| Post-lab summaries | 30 |
| Short-er Communication | 40 |
| Individual participation | 20 |

**Research Project**

The lab will culminate in a research project where you will work in teams to investigate the neuroendocrine basis of behavior in rats. The purpose of this project is to allow you to apply the experimental skills and scientific knowledge you’ll build during the term to carry out experiments to answer a novel neuroscience question. In weeks 8-10, your team will design and conduct experiments to test a hypothesis related to the neural control of reproductive behavior in flies or rats. As part of this project, your team will choose a research question, design an experimental strategy and conduct an experiment to answer your question. Each person will write up the findings in a “Short-er Communication” article format for the journal *Hormones & Behavior*.

**Pre-lab assignments**

We will ask you to do a bit of preparation for labs. Those assignments will be available on Moodle to be submitted before lab starts.

**Post-lab summaries**

The post-lab summaries are designed to help you build the scientific writing skills, including critically analyzing scientific literature, analyzing data and summarizing it with graphs, and writing the different parts of a journal article. *These summaries are due by the next lab session.*

**Brain Practical (Optional)**

We will provide sheep brain sections (physical specimens or photographs) and ask you to identify structures that you observed during the sheep brain dissections. This is a great way to solidify your understanding of neuroanatomy and gain extra credit in the lab.


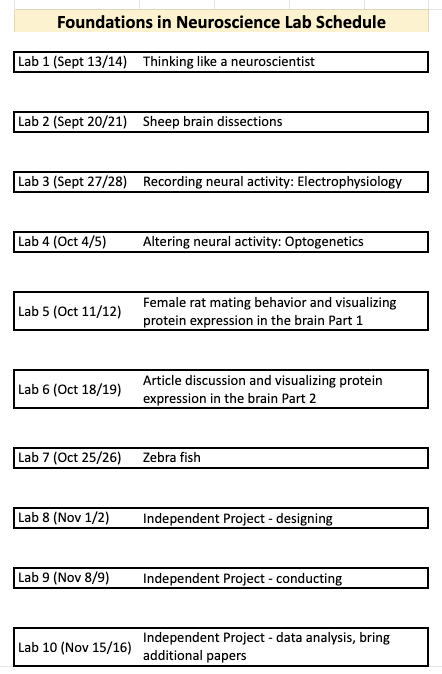

Supplement: Supplemental file 1 [file junejournal_2025_24_1_147378_310525.docx]
