# Supplementary material for: Investigating the Neural Control of Social Behavior in Drosophila ⁠melanogaster Using a Low-Cost Optogenetics System
Source: J Undergrad Neurosci Educ. 2025 Dec 30;24(1):4–14. doi: 10.59390/001c.147378 (PMC13127674; doi:10.59390/001c.147378)
Supplement: Supplemental file 2 [file junejournal_2025_24_1_147378_310526.zip › Supplemental_file_2/Supplemental file 2 contents.pdf]

Supplemental file 2 contents

1. *Behavior examples* contains short video clips of key aggression and courtship behaviors.
2. *Male pair movies* contains the following
   a. Unannotated movies of Lines 1–4 for students to score
   b. Annotated movies of Lines 1-4 for instructor to show
   c. Behaviors_scores.xlsx contains timestamps of aggression and courtship behaviors for each movie.
